# George Macleod

**Published:** 1962

**Authors:** 


### OBITUARY

### GEORGE MACLEOD, M.C., M.A., M.D., D.P.H.

On 2nd October, 1962 George Macleod passed peacefully away at his Clevedon home. By his death this region lost a distinguished man who had long been devoted to the best interests of general practice and of the Clevedon Hospital he loved so well. His kindly humour, forthright common sense and unbounded energy marked him out as a leader of progressive medical thought in this area.

He was born in Scotland 78 years ago and obtained his M.A. Degree at Glasgow in 1905. Four years later he obtained the M.B., Ch.B., (with Honours) and also took the Cambridge D.P.H. in 1912. For his war services in 1914–18 he was awarded the M.C. After the usual resident appointments and further experience as a school medical officer and assistant tuberculosis officer he settled in Clevedon. In addition to his general practice he was appointed M.O.H. for the town—an appointment he occupied until his death. Before the advent of the National Health Service he was the moving spirit in the development of Clevedon's Cottage Hospital, being Chairman of its Committee until 1960.

From 5th July, 1948 he was a very active member of the Southmead Hospital Group Management Committee, and it was largely due to his foresight and initiative that a new Out-Patient Department was established at Clevedon Hospital and The Knoll acquired as a maternity home for the area.

His interest in the British Medical Association is shown by the fact that he was Chairman of the East Somerset Division in 1938–39 and President of the Bath, Bristol and Somerset Branch in 1945–46. He was an ardent supporter of the Bristol Medico-Chirurgical Society and also made time to act as President of the Clevedon Branch of the British Empire Cancer Campaign.

As a relaxation from these many activities he enjoyed his golf and fishing. Throughout his long life George Macleod typified the quiet dignity, unfailing courtesy and sound wisdom of the doctors of his generation.

We tender our deepest sympathy to his widow, his son Alistair—still in practice in Clevedon—and his three daughters.

